# Initial SARS-CoV-2 Vaccination Uptake in a Correctional Setting: Cross-sectional Study

**DOI:** 10.2196/30176

**Published:** 2021-09-28

**Authors:** Justin Berk, Matthew Murphy, Kimberly Kane, Philip Chan, Josiah Rich, Lauren Brinkley-Rubinstein

**Affiliations:** 1 Rhode Island Department of Corrections Cranston, RI United States; 2 Rhode Island Department of Corrections Providence, RI United States; 3 Warren Alpert Medical School Brown University Providence, RI United States; 4 Rhode Island Department of Health Providence, RI United States; 5 Chapel Hill School of Medicine University of North Carolina Chapel Hill, NC United States

**Keywords:** vaccination, COVID-19, incarcerated individuals, correctional facility, public health, pandemic, vaccine, carceral setting, vaccine implementation, correctional staff

## Abstract

**Background:**

The largest outbreaks of COVID-19 in the United States have occurred in correctional facilities, and little is known about the feasibility and acceptability of SARS-CoV-2 vaccine campaigns among incarcerated people.

**Objective:**

The aim of this study was to describe a statewide vaccination program among incarcerated people and staff working in a prison setting.

**Methods:**

Between December 2020 and February 2021, the Rhode Island Department of Corrections (RIDOC) offered the opportunity for SARS-CoV-2 vaccination to all correctional staff and sentenced individuals. Two RIDOC public health educators provided education on the vaccine, answered questions, and obtained consent before the vaccine clinic day for the incarcerated group. All staff received information on signing up for vaccines and watched an educational video that was created by the medical director. Additional information regarding vaccine education and resources was sent via email to the entire RIDOC department.

**Results:**

During this initial campaign, 76.4% (1106/1447) of sentenced individuals and 68.4% (1008/1474) of correctional staff accepted and received the vaccine. Four months after the first vaccine was offered, 77.7% (1124/1447) of the sentenced population and 69.6% (1026/1474) of staff were fully vaccinated.

**Conclusions:**

This study demonstrates the feasibility and efficiency of vaccine implementation in a carceral setting. Education and communication likely played an important role in mitigating vaccine refusals.

## Introduction

The largest outbreaks of COVID-19 in the United States have occurred in correctional facilities [1]. Correctional outbreaks have been shown to contribute to community and statewide spread of infection [2]. The rate of COVID-19 in correctional settings is 5 times that of the general population, and the age-adjusted mortality rate is nearly 4 times higher [3]. Thus, vaccinating individuals who live and work in correctional facilities should be a high priority and is recommended by multiple organizations [4,5]. Despite these recommendations, few states initially prioritized vaccination in correctional settings [6]. Furthermore, vaccine uptake among correctional staff and incarcerated individuals is unknown.

Since the beginning of the pandemic, the Rhode Island Department of Corrections (RIDOC) has collaborated closely with the Rhode Island Department of Health to address COVID-19 with clear testing and isolation procedures, mask wearing, surface sanitation, and ongoing education of staff and incarcerated individuals. Vaccinations were initiated in December 2020.

## Methods

The RIDOC is a unified (combined prison and jail) statewide correctional facility that currently houses approximately 1500 sentenced and 500 awaiting-trial individuals across 6 facilities among a spectrum of security levels, including Minimum Security, Medium Security, Maximum Security, and High Security facilities. The final 2 facilities, Intake facility and Women’s Facility, are jail-like facilities that comprise mostly individuals awaiting trial. The vaccine program initially focused on sentenced individuals (ie, individuals typically housed in a prison). Staff (eg, correctional officers) were concurrently vaccinated at the RIDOC through a parallel vaccine program.

SARS-CoV-2 vaccines were initially offered starting on December 22, 2020, to the sentenced population. By February 5, 2021, the entire sentenced population had received at least one opportunity for vaccination. Second-dose vaccinations for this population were completed by March 5, 2021.

Among incarcerated people, RIDOC leadership prioritized vaccine allocation based on risk factors (as outlined by the Centers for Disease Control and Prevention [CDC] and local Department of Health) and/or security facility. RIDOC nurses administered the vaccine. Two RIDOC public health educators provided education on the vaccine, answered questions, and obtained consent before the vaccine clinic day. All eligible individuals were offered vaccination in this way with the option to accept or defer. Second doses were provided at appropriate time intervals.

Vaccines arrived each week and were distributed in “phases” based on risk factors and logistics. In phase 1, individuals at the highest risk (aged >65 years or >55 years with specific comorbidities) were offered the vaccine. In phase 2, smaller facilities (ie, facilities with a smaller average daily population: Women’s Facility; Minimum, Maximum, and High Security facilities) were offered the vaccine in an attempt to achieve herd immunity in those communities. Phase 3 included the largest remaining security facility—Medium Security as well as sentenced individuals at the Intake facility who were awaiting transfer to one of the sentenced facilities. Phase 4 included all individuals who had previously tested positive for COVID-19 within 90 days and individuals who had initially declined but subsequently accepted. After completion of the four phases, vaccines continued to be offered upon request. A portion of individuals in phase 1 received the Pfizer vaccine, and the rest received the Moderna vaccine.

Among corrections staff, individuals were vaccinated with an opt-in system (signing up via email), prioritizing self-identified high-risk correctional officers (by age and comorbidity) and individuals with direct contact with incarcerated people. During morning “roll call,” all staff received information on signing up for vaccines and watched an educational video that was created by the medical director and made available on the intranet [7]. Additional information regarding vaccine education and resources was sent via email to the entire RIDOC department (Multimedia Appendix 1).

## Results

During the 6-week campaign, a total of 1106 out of 1447 (76.4%) incarcerated individuals and 1008 out of 1474 (68.4%) staff received the vaccine. Among staff, a total of 466 of 1474 individuals (31%) did not opt in for a vaccine during the initial vaccine offering. Table 1 describes the four phases of first-dose vaccination.

**Table 1:** First-dose SARS-CoV-2 vaccination of incarcerated people and correctional staff.

Group		Dates	Offered, N	Vaccinated, n (%)	Declined, n (%)
**Incarcerated people**			1447	1106 (76.4)	341 (23.6)
	Phase 1: Age >65 years, immunocompromised, or age >55 years with comorbidities	Dec 26-29, 2020	143	130 (90.9)	13 (9.1)
	Phase 2: Small facilities (Minimum, Maximum, High, Women’s)	Dec 31, 2020, to Jan 5, 2021	222	143 (64.4)	79 (35.6)
	Phase 3: Medium facility and sentenced individuals awaiting transfer	Jan 13-27, 2021	730	605 (82.9)	125 (17.1)
	Phase 4: All remaining sentenced individuals, including those who had COVID-19 within 90 days	Jan 29 to Feb 5, 2021	352	228 (64.8)	124 (35.2)
**Correctional officers and other staff**					
	Priority to self-reported high-risk individuals and those with direct contact with incarcerated individuals	Dec 22, 2020, to Feb 10, 2021	1474	1008 (68.4)	466 (31.6)

A total of 3 incarcerated individuals and 6 staff members who received their first dose of vaccine opted to not receive their second dose. During this time, “overpulls” (ie, a common 11th dose of vaccine could be pulled from a 10-dose vial) and additional vaccine clinics were offered to incarcerated individuals and staff who ultimately did opt in to receive the vaccine on a rolling basis based on vaccine availability.

Four months after the first vaccine was offered on December 22, 2020, 77.7% (n=1124) of the sentenced population and 69.6% (n=1026) of staff were fully vaccinated. There were no significant vaccine adverse events.

## Discussion

Vaccination was acceptable to individuals in a correctional setting with an acceptance rate of 70% to 75% among both staff and incarcerated people (for comparison, the rate of influenza vaccination uptake at the RIDOC last year was 50.6%). This aligns with necessary immunization rates modeled to achieve herd immunity [8]. More importantly, this is a departure from some concerns of high vaccine hesitancy rates, including a recent CDC publication estimating only a 45% willingness to receive vaccination among incarcerated people [9]. Education and communication likely played an important role in mitigating refusals. Rhode Island, like most other state correctional facilities [10], had COVID-19 outbreaks with fatalities. This may have increased the willingness to get vaccinated. Efforts to increase vaccine uptake have continued.

The high acceptance rate in a correctional setting is particularly relevant given the increased risk of COVID-19–related transmission, disease, and death in this population [3]. The pandemic has substantially affected correctional settings, and the spread of disease in these facilities can catalyze transmission to their surrounding communities [2]. Additionally, both COVID-19 and mass incarceration have disproportionately impacted communities of color [1]. Thus, by vaccinating incarcerated people, policymakers can target a high-risk and marginalized group, decrease community spread, improve equitable allocation to a marginalized group, and potentially reduce the health system costs of neighboring health systems. The successful vaccination of incarcerated individuals and staff in the state of Rhode Island demonstrates the feasibility and efficiency of widespread vaccine programming among those at high risk.

Vaccination of incarcerated people does have unique challenges. Rhode Island was able to coordinate the administration of second doses among the sentenced population without loss to follow-up, but this was in part due to the small size of the state’s population. Additionally, the jail setting offers a greater challenge given the high turnover of the population, often with individuals being released to the community before their second dose is due. While Rhode Island was successful in implementing 2-dose vaccines, strategic implementation of a single-dose vaccine may better align with this unique environment in other larger states, especially for the short-term jailed population.

This vaccine campaign exemplified adherence to public health principles: vaccinate where spread and disease can best be prevented [11]. Correctional settings should remain a priority in vaccination strategies during a pandemic and indeed offer an opportunity to target a high-risk and marginalized population.

## Appendix

Multimedia Appendix 1 RIDOC educational email to staff regarding COVID-19 vaccination.

## Abbreviations

CDC: Centers for Disease Control and Prevention
RIDOC: Rhode Island Department of Corrections
