# Supplementary material for: Initial SARS-CoV-2 Vaccination Uptake in a Correctional Setting: Cross-sectional Study
Source: JMIRx Med. 2021 Sep 28;2(3):e30176. doi: 10.2196/30176 (PMC8483152; doi:10.2196/30176)
Supplement: Multimedia Appendix 1 [file xmed_v2i3e30176_app1.pdf]

Subject: Covid-19 Vaccination Message for Staff
To: Department of Corrections - All Staff

Sending on behalf of Dr. Berk:

The Rhode Island Department of Corrections has gone through a lot in the past year and especially in the past 3 months. I've only been in my role for a short time but have already seen countless examples of resilience across all levels of the staff. Officers have had to adapt to new protocols to keep everyone safe, administrators have had to take on new roles as public health emergency responders, and nurses have across-the-board risen to the occasion to help triage, test, counsel, and treat in addition to all the daily duties regularly performed.

I am lucky to come in to this role at a time where there is a light at the end of the tunnel. We now have rapid BinaxNOW tests that can help quickly identify positive individuals. We have monoclonal antibody treatments in case any high-risk inmate gets sick. **But most importantly: we have a vaccine that is 95% effective**, that has minimal side effects, and is a giant step on the path back to normalcy. The vaccine protects you; it protects your colleagues; and it protects and your family.

I wanted to write to everyone to strongly encourage you to sign up for the Covid-19 vaccine as soon as you are eligible. Alongside RIBCO, we have advocated to ensure that everyone at the ACI will have access to the vaccine. We are extremely fortunate to be among some of the first in line for the Covid-19 vaccine and I hope that everyone takes advantage of this opportunity. This week, I received my second dose of the vaccine (check out the dramatic photo) and could not feel more confident that this will be a game changer in the fight against coronavirus.

For those who have not had Covid (and therefore have some natural immunity), we plan to offer vaccine to **all staff within the next two weeks.**

For those who previously tested positive for Covid: you will still be eligible and be offered vaccine before March at the latest.

The vaccine is EFFECTIVE and it is also SAFE. There are some side effects: a sore arm, headache, and muscle aches only for 1 – 2 days. There are **no risks of infertility** or catching the virus. For more info, read here.

Whether you have risk factors or are young and healthy: the vaccine can ensure you do not get sick and can help ensure you don't contribute to the spread of disease. For those waiting to see how others respond, **you're in safe company: OVER 30 MILLION PEOPLE have safely received the vaccine** and the number is growing.

ATTACHED is more information and answers to some frequently asked questions regarding the vaccine. Check out the **video created for RIDOC staff** for more information. If you have further concerns or questions, please feel free to reach out to me directly any time and I will provide answers to the best of my ability.

Thank you for all that you do. Stay safe!

Justin Berk, MD MPH MBA
Director, Medical Services
Rhode Island Department of Corrections
